# Geochemical and Dietary Drivers of Mercury Bioaccumulation in Estuarine Benthic Invertebrates

**DOI:** 10.1021/acs.est.2c03265

**Published:** 2022-06-30

**Authors:** Sofi Jonsson, Van Liem-Nguyen, Agneta Andersson, Ulf Skyllberg, Mats B. Nilsson, Erik Lundberg, Erik Björn

**Affiliations:** † Department of Environmental Science, Stockholm University, SE-106 91 Stockholm, Sweden; ‡ Department of Chemistry, Umeå University, SE-901 87 Umeå, Sweden; § Umeå Marine Sciences Centre, Umeå University, SE-910 20 Hörnefors, Sweden; ∥ Department of Ecology and Environmental Science, Umeå University, SE-901 87 Umeå, Sweden; ⊥ Department of Forest Ecology and Management, Swedish University of Agricultural Sciences, SE-901 83 Umeå, Sweden

**Keywords:** monomethylmercury, inorganic divalent mercury, benthic food webs, mercury uptake, biomagnification, Baltic Sea

## Abstract

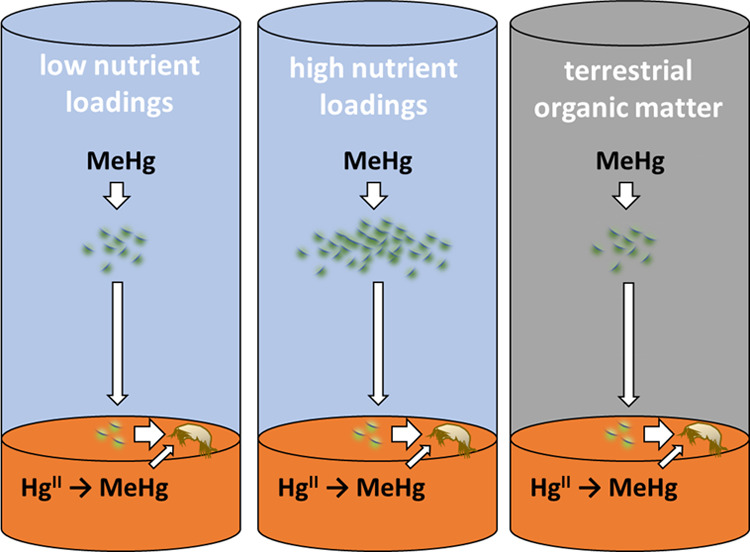

Sediments represent the main reservoir of mercury (Hg) in aquatic environments and may act as a source of Hg to aquatic food webs. Yet, accumulation routes of Hg from the sediment to benthic organisms are poorly constrained. We studied the bioaccumulation of inorganic and methylmercury (Hg^II^ and MeHg, respectively) from different geochemical pools of Hg into four groups of benthic invertebrates (amphipods, polychaetes, chironomids, and bivalves). The study was conducted using mesocosm experiments entailing the use of multiple isotopically enriched Hg tracers and simulation of estuarine systems with brackish water and sediment. We applied different loading regimes of nutrients and terrestrial organic matter and showed that the vertical localization and the chemical speciation of Hg^II^ and MeHg in the sediment, in combination with the diet composition of the invertebrates, consistently controlled the bioaccumulation of Hg^II^ and MeHg into the benthic organisms. Our results suggest a direct link between the concentration of MeHg in the pelagic planktonic food web and the concentration of MeHg in benthic amphipods and, to some extent, in bivalves. In contrast, the quantity of MeHg in benthic chironomids and polychaetes seems to be driven by MeHg accumulation via the benthic food web. Accounting for these geochemical and dietary drivers of Hg bioaccumulation in benthic invertebrates will be important to understand and predict Hg transfer between the benthic and the pelagic food web, under current and future environmental scenarios.

## Abstract 2

A mesocosm study revealing bioaccumulation pathways of mercury into amphipods, polychaetes, chironomids, and bivalves.

## Introduction

Monomethylmercury (MeHg) is neurotoxic and bioaccumulates and biomagnifies in aquatic food webs. While high concentrations of MeHg are primarily reached in pelagic organisms occupying high trophic positions (including predatory fish and marine mammals), sediments are the main abiotic reservoir of mercury (Hg) in aquatic environments.^1^ Sediments also provide conditions favorable for biotic methylation of inorganic divalent Hg (Hg^II^), the primary form of Hg in sediments, to MeHg. The MeHg produced in, or deposited to, sediments may then be bioaccumulated by benthic invertebrates and subsequently transferred to the pelagic food web by foraging pelagic predators.^2−6^ A detailed understanding regarding uptake routes for Hg in benthic invertebrates is, however, missing.

Benthic organisms can accumulate Hg^II^ and MeHg through adsorption from the sediment pore water or through the diet.^4^ Both Hg^II^ and MeHg have a strong affinity to particles, which limits their availability for biological uptake directly from the pore water. The bioavailability of Hg^II^ and MeHg in the dissolved phase is further controlled by the type of ligand they are bound to.^7,8^ Diet intake is the main MeHg exposure pathway for organisms at higher trophic levels in the benthic food web and may involve both detritus (i.e., dead organic particulate matter) and living organisms originating from the sediment or the pelagic zone. Diet analysis of benthic fauna is, however, challenging, and the diet (and Hg accumulation routes) of most benthic invertebrates remains largely unknown.^9^ The organic fraction of sediments is a complex mixture of dissolved organic matter (DOM), detritus, and a benthic food web composed of bacterial and phytobenthic communities, as well as macrofauna such as amphipods, chironomids, bivalves, and polychaetes (the invertebrates sampled in this study).^10,11^ This sediment heterogeneity, with respect to the quality of organic matter as a carbon source for benthic organisms, may play an important role in the transfer of Hg to benthic organisms. For example, Cremona et al.^12^ found that freshwater macroinvertebrates in a lake with extensive macrophyte beds utilized carbon and were exposed to MeHg, mainly from epi- and macrophytes. The potential role of available carbon sources for the accumulation of Hg via the diet also means that uptake routes of Hg may change over the season and with changing environmental conditions if the quantity and/or quality of the organic matter deposited is altered. This has, for example, been shown in the freshwater system studied by Cremona et al.,^12^ where particulate organic matter only contributed to the macroinvertebrates’ diet in mid-summer when suspended particles contained a larger fraction of fresh algae.

Linking the concentration of Hg stored in sediments to the concentration of Hg accumulated in different taxonomical groups of benthic invertebrates requires novel approaches taking into account the availability of Hg, the quality of organic matter as a carbon source for benthic organisms, and trophic transfer processes. To address these aspects, we evaluated previously published and unpublished data on the bioaccumulation of Hg into benthic invertebrates from two mesocosm studies where different geochemical Hg pools and loading regimes of nutrients and terrestrial organic matter were simulated.^13−16^ Briefly, 12 mesocosms were utilized, and one set of isotopically enriched Hg^II^ and MeHg tracers were injected into intact sediment cores (with a diameter of 0.65 m and a depth of ∼0.20 m) and a second set (with differently labeled Hg^II^ and MeHg tracers) were added to the pelagic zone (4.7 m high brackish water column). These isotopically enriched Hg tracers represent different geochemical pools of Hg, defined as Hg with different chemical forms and/or environmental compartment localization. We have previously demonstrated that MeHg and Hg^II^ loadings deposited to the sediment surface from the water column were more available to benthic invertebrates than MeHg stored or formed in the sediment from pools representing previously deposited Hg.^13^ The aims of the study presented here were to unravel (i) how the bioaccumulation of Hg^II^ and MeHg in different invertebrate taxa (amphipods, chironomids, bivalves, and polychaetes) was controlled by the vertical distribution of Hg tracers, feeding behavior of the organism, and amount and type of deposited organic matter; and (ii) to what extent MeHg bioaccumulation in these benthic invertebrates was channeled via the pelagic food web and via the benthic food web. These research aims were explored using observed accumulation patterns of Hg^II^ and MeHg tracers, stable carbon and nitrogen isotope signatures, and known differences in carbon utilization among collected invertebrates.

## Experimental Section

Two separate mesocosm experiments were conducted in 2010, one for 8 weeks from September to October (referred to as M1-NP_low_, M1-NP_high_, M1-TM) and one for 4 weeks in February (hereon referred to as M2). Moderate additions of nutrients (NO_3_^–^, NH_4_^+^, and PO_4_^3–^) were made to M1-NP_low_, simulating present-day spring bloom conditions in the Bothnian Sea (Table S1). Two additional treatments with high nutrient addition or addition of humic soil extract (M1-NP_high_ and M1-TM) were made to conceptually simulate a eutrophication and an increased terrestrial runoff (climate change) scenario, respectively. For M2 systems, moderate nutrient additions, similar to M1-NP_low_, were made during weeks 1–2 of the experiment, and higher additions, similar to M1-NP_high_, were made during weeks 3–4 of the experiment. Detailed information concerning the mesocosm setup, sampling, and analyses of water, sediment, and biota are provided elsewhere^13−15^ (analysis of Hg in biota is summarized in the Supporting Information). In brief, intact sediment cores (diameter of 0.65 m, depth of 0.2 m) were collected by divers in the Öre Estuary, northern Bothnian Sea, Baltic Sea, Sweden (Figure S1), at a water depth of 5–7 m. Isotopically enriched Hg tracers, β-^200^HgS(s), ^201^Hg-NOM, and Me^198^Hg-NOM, were injected into the sediment cores at a depth of 0.5 cm in the M1 systems. In M2, the sediment tracers, β-^201^HgS(s), ^200^Hg-NOM, and Me^198^Hg-NOM, were injected at a sediment depth of 1 cm. The added tracers were synthesized to represent dominant chemical forms of MeHg and Hg^II^ encountered in marine sediments, i.e., Hg^II^ and MeHg bonded to thiol functional groups in natural organic matter (NOM) and Hg^II^ in the form of the dominant mineral phase metacinnabar (β-HgS). The sediment cores were then immersed in 5 m high, temperature- and light-controlled mesocosm systems with brackish seawater at the Umeå Marine Sciences Centre, Norrbyn, Sweden. Two tracers were added to the water column: ^204^Hg^II^(aq) and Me^199^Hg(aq), simulating recent terrestrial and atmospheric inputs of Hg^II^ and MeHg, respectively. In the M1 systems, the two water-phase tracers were added at the beginning of the experiment, and biota (seston of different size fractions and benthic invertebrates) was collected 8 weeks later. In the M2 systems, the water-phase tracers were added at the beginning and after 2 weeks of the experiment, and biota was collected after 4 weeks of the experiment (i.e., 2 weeks after the second addition of tracers to the water phase).

Benthic invertebrates were collected by sieving the sediment at the end of the experiments. Collected invertebrates were left in filtrated seawater overnight to allow for gut depuration. The biota was then sorted, freeze-dried, homogenized, and analyzed for isotope selective concentrations of total Hg and MeHg and the δ^13^C and δ^15^N isotopic signatures. Before the biota was freeze-dried and homogenized, individual invertebrates from the same taxonomical group and mesocosm were pooled. Due to insufficient data on the δ^13^C signature for end members (e.g., phytoplankton and bacteria) present in the mesocosm systems, we primarily used the δ^13^C signatures in a qualitative approach to support differences or similarities in organic matter sources among invertebrates. We did not characterize the taxonomical composition of collected amphipods, bivalves, and polychaetes but the zoobenthic community has been characterized in the Öre Estuary at several sites close to where the sediment was collected for this study (Supporting Information, Figure S1 and Table S2, data compiled from 2010, Swedish Meteorological and Hydrological Institute). The zoobenthic community of amphipods, bivalves, and polychaetes at these sites was dominated by the amphipod *Monoporeia affinis*, the polychaete *Marenzelleria*, and the bivalve *Macoma balthica* (Supporting Information, Tables S2 and S3). In the discussion below, we assume the same species to dominate the amphipods, bivalves, and polychaetes collected from our systems.

No animals were added to these experiment systems; all data originate from benthic invertebrates already present in the intact sediment cores (diameter of 0.65 m) at the time of sampling. The number of organisms collected was thus limited, and the number of animals in the different systems varied (Supporting Information, Table S4). Our approach, however, allowed us to collect data on Hg bioaccumulation from benthic invertebrates that have not been stressed by relocation and that remained in sediments kept undisturbed until the end of the experiment with respect to the redox cline and the vertical distribution of organic matter. Further, organic matter was continuously deposited from the pelagic zone to the surface of the sediment during the course of the experiments.

The availability of ambient Hg and added Hg tracers to benthic invertebrates is presented as the Hg^II^- and MeHg-biota to sediment accumulation factor (BSAF) (ratio between concentrations in biota and sediment in pmol g^–1^ d.w., Supporting Information, Tables S5 and S6). The MeHg and Hg^II^ concentrations of all added tracers and ambient Hg were measured in the top 1.5 cm (M1) or 2 cm (M2) of sediment. Calculated BSAF values for tracers in M2 were corrected for differences in sediment sampling depth between systems by recalculating the average concentration of tracer to the top 1.5 cm, assuming all sediment tracer amount remained at the injected depth (1 cm) ± 0.5.

Statistical analysis was conducted using JMP-Pro (version 15.0.0, SAS Institute Inc.) software. Differences in %MeHg, the isotopic signature of δ^13^C and δ^15^N, Hg^II^-BSAFs, and MeHg-BSAFs were tested by two-way analysis of variance (ANOVA) using the invertebrate group as independent variables. Differences in MeHg-BSAFs were also tested by ANOVA using M1-treatment (NP_low_, NP_high_, and TM) and invertebrate group as independent variables. For the statistical analysis, all BSAFs were log-transformed. Tukey’s test was used to test for differences among groups. The null hypothesis (no difference among treatments) was verified by a *p*-value ≥ 0.05.

Previously published data evaluated further in this work include ancillary parameters from M1 and M2 (e.g., primary production, nutrient levels, and the concentration of MeHg in seston)^13−15^ and MeHg-BSAFs for the invertebrates from M1-NP_low_.^13^^13^ Additional ancillary parameters monitored have been presented previously^13−15^ and are summarized in Supporting Information, Tables S1 and S7.

## Results and Discussion

### Bioaccumulation of Hg^II^ and MeHg from Different Geochemical Hg Pools under Different Treatment Regimes

**Figure 1:**
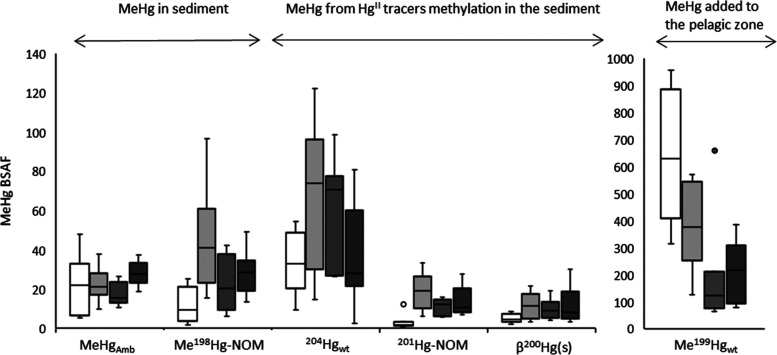
Box plots showing 25th, 50th, and 75th percentiles (horizontal bars), 1.5 interquartile ranges (error bars), and maximum outlier (open circles) for methylmercury biota sediment accumulation factor (MeHg-BSAF) for ambient Hg and the different Hg tracers added in M1 experiments (M1-NP_low_,^13^^13^ M1-NP_high_, and M1-TM). The different bars show BSAF for amphipods (n=8, white bars), polychaetes (n=9, light gray bars), chironomids (*n* = 7, intermediate gray bars), and bivalves (*n* = 9, dark gray bars).

We applied three experimental treatments to investigate if the altered amount or type of organic matter deposited to the sediment impacts the bioaccumulation of Hg^II^ and MeHg from different geochemical Hg pools in benthic invertebrates. The different treatments were moderate (M1-NP_low_) and high (M1-NP_high_) loading of nutrients (NP) and high loading of terrestrial organic matter (M1-TM). For M2, a moderate nutrient loading was applied during the first half of the experiment and a high loading during the second half. These treatments resulted in different production of autochthonous organic matter in the pelagic zone (Table S1) and ultimately variable deposition rates of autochthonous and allochthonous organic matter to the sediment (Figure S5 in Jonsson et al. 2017). The overall trends in bioaccumulation of MeHg from the different added tracers in the M1-NP_low_ treatment, simulating present-day spring bloom conditions in the Bothnian Bay, have been discussed elsewhere.^13^ Briefly, bioaccumulation of tracers added to the mesocosms sediment depended on the vertical distribution of the tracer (tracers deposited from the water column to the sediment surface vs tracers injected at a larger sediment depth) as well as on the chemical form of the tracers. Tracers added to the water column were bioaccumulated to a larger extent than those injected into the sediment, and tracers added as MeHg were bioaccumulated more than MeHg formed in situ from Hg^II^ tracers added to the sediment. We observed the same relative bioaccumulation pattern of the different geochemical pools of MeHg in the M1-NP_high_ and M1-TM mesocosms (Figures 1 and S1; MeHg-BSAF for the MeHg_wt_ tracer greater than MeHg-BSAF of the other tracers (*p* < 0.05); median MeHg-BSAF for Hg_wt_ exceeding median MeHg-BSAF for NOM-Hg_sed_^II^ and β-HgS_sed_ (*p* < 0.05) in M1-NP_high_; median MeHg-BSAF for Hg_wt_ exceeding median MeHg-BSAF for NOM-Hg_sed_^II^ (*p* < 0.05) in M1-TM). The same trends were also observed in M2 mesocosms (Supporting Information, Figure S2), where moderate to high levels of nutrients were added. The high bioaccumulation of the MeHg tracer added to the water column (MeHg_wt_) observed for all invertebrates and experimental treatments was likely due to that the tracer was incorporated into autochthonous OM of high quality as an energy source for consumers (e.g., algal biomass) before being deposited to the surface of the sediment. In contrast, MeHg added to, or produced in, the sediment likely was associated with OM of lower quality. It has previously been demonstrated that the MeHg_wt_ tracer accumulated to a higher degree than the other tracers in pelagic seston, which provide further support for the incorporation of the MeHg_wt_ tracer into autochthonous carbon sources.^13,15^ The consistent pattern observed in all four sets of mesocosms (M1-NP_low_, M1-NP_high_, M1-TM, and M2) supports the importance of MeHg deposited or newly formed at the surface of the sediment (represented by the Hg_wt_ tracer) for bioaccumulation of MeHg into benthic invertebrates across systems with different nutrient and terrestrial organic matter loading regimes. The results show that the vertical distribution of Hg in the sediment is more important than variation in the amounts of autochthonous and allochthonous organic matter deposited to the sediment (within the ranges investigated in this study) as a driver for Hg accumulation in benthic invertebrates.

Although the same relative bioaccumulation pattern of added tracers was observed in M1 and M2, the MeHg-BSAF values were not always in the same range for all tracers. For the MeHg tracer added to water, we observed higher MeHg-BSAFs in M2 systems than in M1 systems. In M2 systems, MeHg_wt_ and Hg_wt_ were added twice during the four-week experiment compared to only once at the beginning of the eight-week experiment in the M1 systems. These differences can explain the higher MeHg-BSAFs from MeHg_wt_ in M2 (Figure S2). The sediment Hg tracers were injected at 1 or 0.5 cm sediment depth in M2 and M1 systems, respectively. The MeHg-BSAFs for MeHg originated from these tracers were, however, similar in M2 and M1 systems, demonstrating that the exact depth (0.5 or 1 cm) of MeHg localized well beneath the sediment–water interface caused only minor differences in bioaccumulation. It is worth noting that this also was the case for organisms (e.g., *Marenzelleria*) known to burrow down to depths as large as 50 cm.^17^ Different availability of contaminants due to their localization depth in sediments is in line with earlier studies. Josefsson et al.,^18^ e.g., showed higher bioaccumulation in amphipods and polychaete sp. (sampled in the same estuary as the sediment and biota used in our mesocosm experiments) of polychlorinated biphenyls (PCBs) and polybrominated diphenyl ethers (PBDEs) mixed into the sediment at a sediment depth of 2 cm compared to 5 or 10 cm.^18^ For the estuarine amphipod *Leptocheirus plumulosus*, Taylor et al.^19^ further demonstrated greater accumulation of MeHg and Hg^II^ via phytoplankton deposited onto the surface of the sediment in 0.1 L microcosm systems compared to via phytoplankton mixed into the sediment phase. In contrast to previous studies, we studied the bioaccumulation of Hg in relatively intact sediment systems where the heterogeneity of the sediment with respect to redox cline and vertical distribution of organic matter relative to natural conditions were maintained. Thus, we are able to show the heterogeneity of Hg bioavailability within the top cm of sediments and are able to provide accumulation rates that are more likely to be representative of the bioaccumulation rates in natural systems.

Both Hg^II^ and MeHg bioaccumulate in aquatic organisms,^20^ however, not necessarily through the same pathways. In M1 systems, MeHg-BSAF in benthic invertebrates ranged from 0.6 to 1200 for added Hg tracers and between 5 and 48 for ambient MeHg, whereas the Hg^II^-BSAF for added Hg tracers and ambient Hg were in the range of 0.009–4 and 0.01–90, respectively (Supporting Information, Figures S2 and S3). Bioaccumulation (i.e., BSAF > 1) was observed for MeHg from all Hg tracers and ambient MeHg, whereas bioaccumulation of Hg^II^ was only consistently observed for the added Hg_wt_ and MeHg_wt_ (i.e., bioaccumulation of Hg^II^ from demethylated MeHg_wt_) tracers. These observations are in line with earlier work showing that both Hg^II^ and MeHg can bioaccumulate in, e.g., phytoplankton^20^ but that MeHg bioaccumulates and magnifies to a greater extent.^21^ Interestingly, the Hg^II^-BSAF values for tracers added as Hg^II^ followed the same trend as the MeHg/Hg^II^ values (used as a proxy for the net methylation) of the same tracers; Hg_wt_ > Hg-NOM_sed_ > β-HgS_sed_,^13^ and no differences were noted between treatment regimes. The difference in availability of the Hg^II^ tracers for net methylation has been explained by a combination of thermodynamic stability of their solid and adsorbed phase Hg^II^ species (controlling the relative solubility of the different Hg^II^ tracers) and compartment localization of the tracers.^13^ If assuming the accumulation of Hg^II^ in benthic invertebrates occurs mainly through the uptake of dissolved Hg^II^ from the pore water, thermodynamic stability and compartment localization of the Hg tracer species likely also explain the different Hg^II^-BSAFs observed for the added Hg^II^ tracers. Although Hg^II^ is not as efficiently transferred to organisms higher up in the food web, in comparison to MeHg, Hg^II^ adsorbed on organisms preyed upon could potentially be methylated in the gut of their predator. Recent work has, for example, identified the hgcA genes (one of the Hg methylating genes) in the gut microbiome of copepods from the Baltic Sea.^22^ The role of gut Hg methylation for the pool of MeHg accumulating in aquatic food webs, however, remains to be supported.

### Bioaccumulation of MeHg into Different Invertebrates

**Figure 2:**
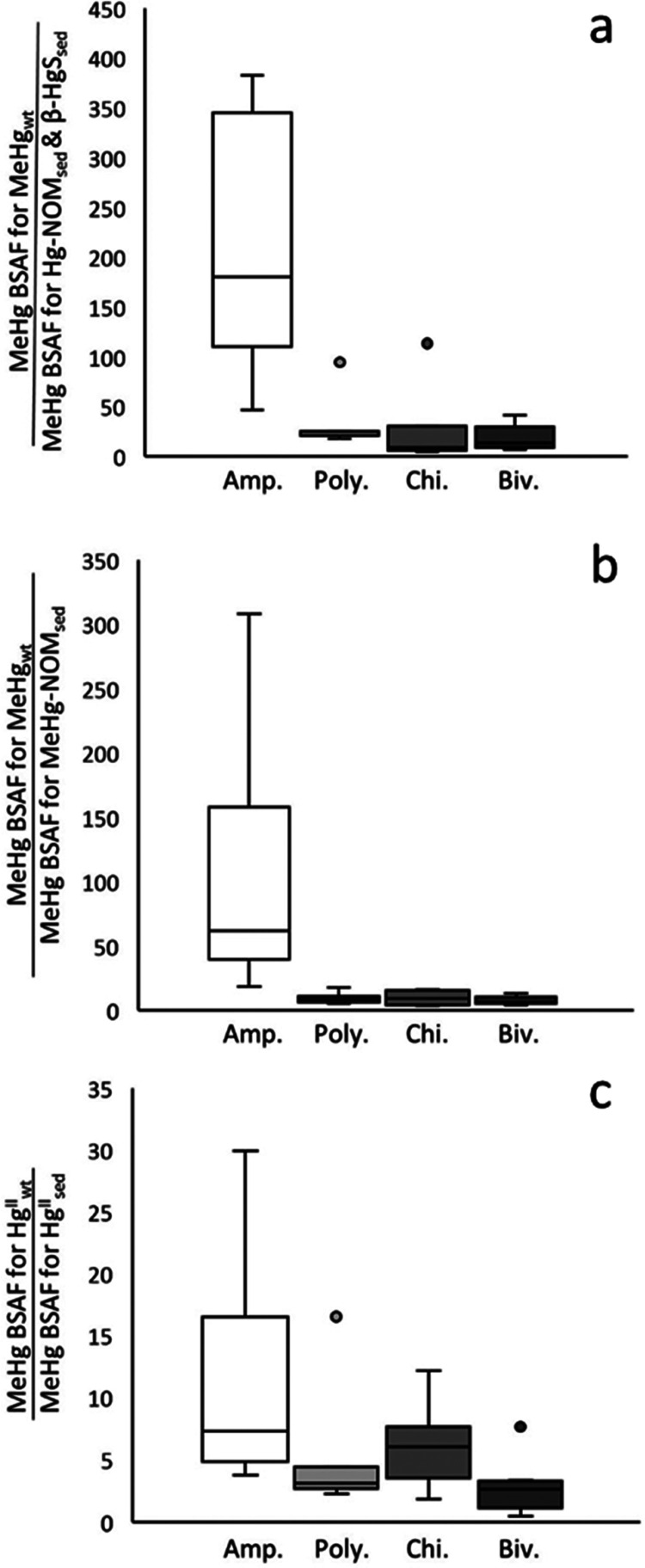
Box plots showing 25th, 50th, and 75th percentiles (horizontal bars), 1.5 interquartile ranges (error bars), and maximum outlier (open circles) for MeHg-BSAF ratios for (a) MeHg tracer added to the water and MeHg formed from inorganic tracers injected into the sediment (MeHg_wt_ vs Hg-NOM_sed_ and β-HgS_sed_), (b) MeHg tracer added to the water and MeHg tracers injected into the sediment (MeHg_wt_ vs MeHg-NOM_sed_), and (c) MeHg formed from inorganic tracers added to the water and inorganic tracers injected to the sediment (Hg_wt_ vs Hg-NOM_sed_ and β-HgS_sed_) in M1 systems. The different bars show data for amphipods (Amp.), polychaetes (Poly.), chironomids (Chir.), and bivalve (Biv.).

Although all invertebrates accumulated MeHg added to the water column (MeHg_wt_) to a higher degree than MeHg injected to, or formed in, the sediment, there were systematic differences among the different invertebrate taxons (Figure 1). On average, the MeHg-BSAF of MeHg_wt_ for amphipods were 100 and 210 times higher than of MeHg added to (NOM-MeHg_sed_) or formed in (NOM-Hg_sed_^II^, β-HgS_sed_) the sediment, respectively (Figure 2a,b). For polychaetes, chironomids, and bivalves, the MeHg-BSAF of MeHg_wt_ was, however, only, on average, 18–32 and 7.7–9.6 times higher than MeHg-BSAF of MeHg for the two sets of tracers added to the sediment, respectively. The MeHg-BSAF of MeHg formed from the Hg_wt_ tracer deposited to the surface of the sediment was more available than MeHg formed from tracers injected at a depth of 5 mm. These differences were similar for all invertebrates (Figure 2c). Our results suggest that the amphipods, under all treatment regimes, to a higher degree accumulated MeHg from the pelagic food web than the other benthic invertebrates. Even if all invertebrates preferably feed on autochthonous OM, with high quality, differences in feeding behavior among the invertebrate organisms could explain the systematic differences in MeHg accumulation. Different feeding behaviors among the invertebrates are also supported by earlier work showing, e.g., a more pelagic diet of *Monoporeia affinis*, in comparison to the polychaete *Marenzelleria*.^23,24^

**Figure 3:**
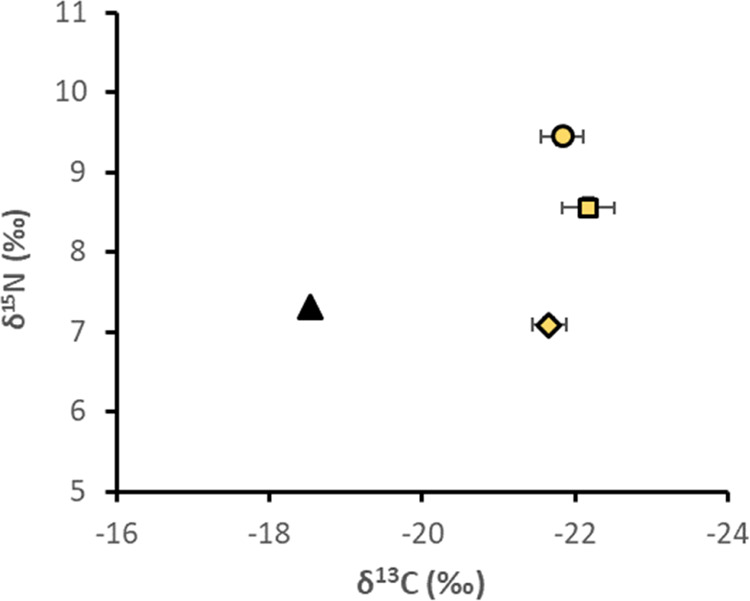
Average δ^13^C and δ^15^N isotope signatures (‰) in amphipods (▲, *n* = 1), polychaetes (O, *n* = 9), chironomids (□, *n* = 5), and bivalves (◊, *n* = 9) in M1. Whiskers show ± 1 SE.

To gain further insights into differences in feeding behavior among the benthic invertebrates, we measured carbon and nitrogen isotope composition in the organisms. Such measurements are commonly used to specify carbon feeding sources (δ^13^C) and trophic position (δ^15^N) of organisms.^25^ The analysis of isotopic niches (using the δ^13^C and δ^15^N signatures), however, relies on a number of assumptions and is not always straightforward. The δ^13^C signature of several carbon sources may overlap, and the δ^15^N signature may be altered by other processes than trophic enrichment. The δ^13^C signature for bivalves, chironomids, and polychaetes collected from our systems was in the range of −23.4 to −20.5‰ and no statistical difference was found among the organism groups (*p* > 0.05; Figure 3). Due to the limited amount of biomass of amphipods collected from the mesocosms, only one of the samples (from a M1-TM mesocosm) could be analyzed for stable C and N isotopes. The δ^13^C signature of −18.5‰ observed in this sample was higher than the δ^13^C signature in bivalves, chironomids, and polychaetes, thus supporting a different diet composition of the amphipod (as also indicated by the MeHg-BASF values).

**Figure 4:**
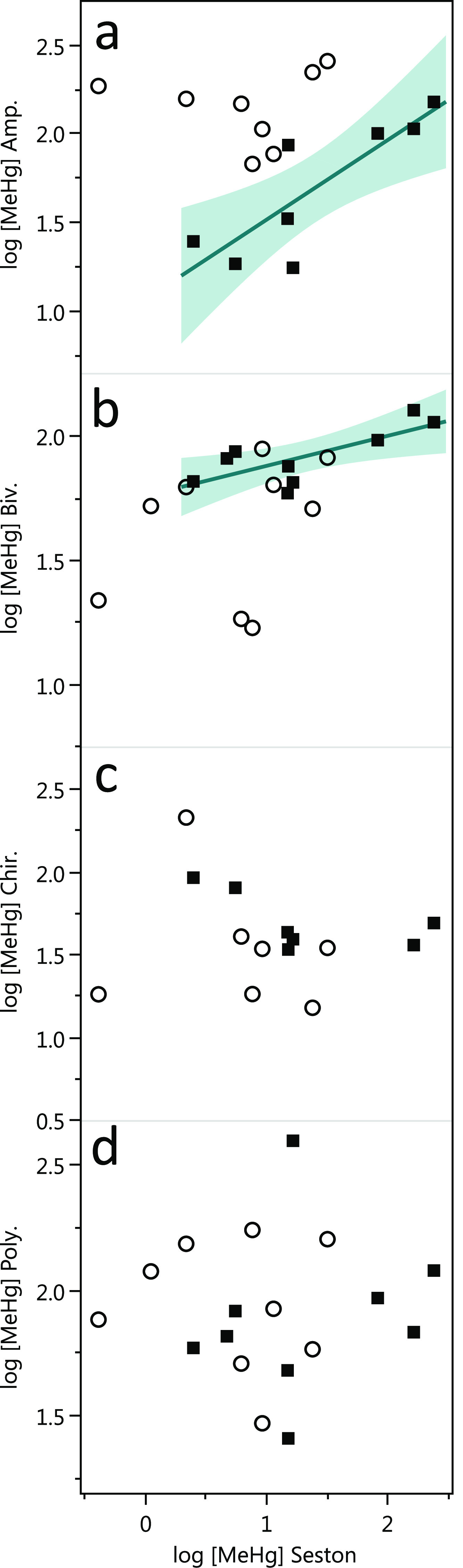
Concentrations of ambient MeHg (filled squares) and MeHg_wt_ tracer (empty circles) accumulated in (a) amphipods (Amp.), (b) bivalves (Biv.), (c) chironomids (Chir.), and (d) polychaetes (Poly.) as a function of the MeHg concentration in seston^15^ (average of collected size fractions, 50–300 μm) from M1 systems. Solid lines show and shaded area shows the linear fit and the confidence of fit, respectively, for ambient MeHg for statistically significant correlations (*p* < 0.05, a, ambient MeHg: *R*^2^ = 0.70 and *p* = 0.01; b, ambient MeHg: *R*^2^ = 0.56 and *p* = 0.02).

When comparing the polychaetes with the bivalves and chironomids collected from our mesocosm systems, the relative accumulation of MeHg from the different geochemical pools did not differ significantly (Figure 1). Further, they all had similar, and compared to the amphipod a lower, δ^13^C signature (Figure 3), suggesting that the polychaetes, chironomids, and bivalves have a similar source of carbon. However, the polychaetes had, in comparison to the bivalves and chironomids, a higher %MeHg of ambient Hg and of MeHg originating from methylation of the three Hg^II^ tracers added or deposited to the sediment (Table S8). These results suggest that the polychaetes either accumulated MeHg more efficiently or digested material with a higher %MeHg. The polychaetes collected from the sediments in our system were primary *Marenzelleria*, the most abundant polychaete in the estuary sampled (Table S2). Gut content analysis of benthic invertebrates is challenging and there are, to our knowledge, no current studies that in detail describe the diet composition of adult *Marenzelleria* Polychaetes.^9^ However, selective feeding is common for polychaetes, and particle size selectivity has been demonstrated for *Marenzelleria viridis*.^26^ Isotopic niche analysis of benthic invertebrates collected from stations in the north-western Baltic proper has also suggested the polychaete *Marenzelleria arctica* to occupy a different niche than the dominating native species (the bivalve *Macoma balthica* and the amphipod *Pontoporeia femorata*).^25^ In line with this field study, we observed an enriched δ^15^N signature for the polychaetes, in comparison to the bivalves and amphipods (*p* < 0.05; Figure 3). Karlson et al.^25^ suggested microbial recycling of N in the benthic food web and a greater fractionation within the polychaete due to a higher demand for N (as also seen in our study, Figure S4) to explain the enriched δ^15^N signature. Enriched δ^15^N signature for the polychaetes could thus imply that the polychaetes, to a higher degree than the other invertebrates, accumulated MeHg incorporated (and potentially enriched) in the benthic food. In line with this observation, no direct correlation was observed between ambient MeHg concentration in seston and polychaetes (or chironomids), while such a correlation was found for bivalves (Figure 4, discussed further below).

### Trophic Transfer of MeHg from Pelagic Plankton to Benthic Invertebrates

We investigated if the MeHg accumulation in benthic invertebrates was driven by MeHg concentrations in pelagic seston (continuously depositing to the sediment surface as detritus). Concentrations of MeHg in amphipods, chironomids, bivalves, and polychaetes collected from M1 were plotted as a function of seston MeHg concentrations (Figure 4). For ambient MeHg, the average concentration in seston fractions correlated positively with the concentration in amphipods (*r*^2^ = 0.7, *p* < 0.05, Figure 4). Also for bivalves, a significant correlation, yet with a smaller slope, was observed (*r*^2^ = 0.56, *p* < 0.05). No correlation was observed between the concentrations of ambient MeHg in seston and in chironomids and polychaetes nor between the concentration of MeHg_wt_ tracers in seston and any of the benthic invertebrates. The lack of correlation for MeHg_wt_ could be due to the fact that MeHg_wt_ concentrations in seston (except for one data point) spanned within a smaller range (1.1–31 pmol g^–1^ d.w.) than ambient MeHg (2.5–240 pmol g^–1^ d.w.).^15^ The results for ambient MeHg, however, suggest a direct pelagic–benthic food-web coupling for the bioaccumulation of MeHg in amphipods, and potentially to some extent in bivalves, and that MeHg concentration in plankton is an important controlling factor for MeHg concentrations in these benthic organisms. In contrast, the coupling between MeHg accumulating in plankton and in chironomids and polychaetes appears to be weaker. Altogether, the accumulation patterns of Hg tracers combined with δ^13^C (amphipods < bivalves, chironomids, polychaetes) and δ^15^N (polychaetes > chironomids > bivalves, amphipods) signatures suggest that the accumulation of MeHg in benthic invertebrates from the pelagic food web relative from the benthic food web decreased in the order: amphipods > bivalves > chironomids > polychaetes.

### Environmental Implications

A better understanding of the interactions between the pelagic and benthic compartments is warranted to understand to what extent, and under what conditions, Hg in estuarine sediments is transferred to pelagic food webs. First, knowledge of uptake routes of Hg into benthic invertebrates, including effects caused by the vertical distribution and quality/availability of Hg and OM, is important to predict timeframes for the “burial” of Hg in sediments (i.e., the sediment depth below which the Hg pool is not bioaccumulated). With our novel experimental approach (entailing the use of 2000 L mesocosm tanks with intact sediment cores and the use of multiple Hg tracers), we demonstrate a strong difference in bioaccumulation of Hg even within the top millimeters to centimeter of sediments. As this vertical heterogeneity is normally not accounted for when examining the correlation between Hg concentrations in surface sediments (typically defined as the top centimeter or more of sediments) and pelagic organisms in field studies,^27,28^ important benthic–pelagic coupling mechanisms may previously have been overlooked.

Second, pelagic drivers of the composition and abundance of the benthic food web, as well as feeding habits of specific invertebrate taxa, need to be considered. The Baltic Sea has undergone several regime shifts in recent times that have led to alterations in the benthic communities.^29^ Rapidly declining population densities of the amphipod *Monoporeia affinis* in the late 1990s has, for example, been linked to a concurrent decline in pelagic primary production.^23,24^ Substantial losses of benthic fauna have also occurred due to the development of anoxic bottom waters.^30^ Future pelagic–benthic couplings in the Baltic sea, and likely many coastal regions, will depend on the combined effect of nutrient inputs and climate change.^29^ While increased nutrient loads lead to increasing loads of OM to the seafloor, a warmer climate results in more internal recycling of the OM in the pelagic zone and thus less sedimentation of OM to the benthic zone. As we have shown in earlier work,^15^ increased loading of terrestrial organic matter may result in enhanced accumulation of MeHg in zooplankton due to shifts in the base of the pelagic food-web structure. The study reported here suggests that such alterations will lead to a direct enhanced concentration of MeHg in amphipods and to a lesser extent also in bivalves, both important dietary sources for pelagic fish such as sprat (*Sprattus sprattus*) and herring (*Clupea harengus*).^31^ In contrast, such a direct link does not seem to exist (at least within the timeframe of our experiments) for benthic invertebrates that to a larger extent utilize sediment OM, such as the polychaetes. The accumulation of MeHg in these types of invertebrates is more likely driven by the total amount of MeHg (regardless of its origin) available for incorporation in the benthic food web and by subsequent trophic transfer processes. Taking these processes into account will be important to advance our understanding and enabling predictions of Hg transfer from the benthic to the pelagic food web under current and future environmental scenarios.

## Supporting Information Available

Brief description of the analysis of Hg in biota; map showing sampling location and location of monitoring stations for benthic invertebrate abundance presented in Table S2 (Figure S1); average MeHg-BSAF and Hg^II^-BSAF in benthic invertebrates for tracers (Figure S2) and ambient Hg (Figure S3); percent carbon and nitrogen (Figure S4); pelagic biological productivity (Table S1); abundance of benthic fauna (Table S2); taxonomical information of zoo benthos (Table S3); number of individuals collected from the experiments and average ambient MeHg concentration (Table S4); concentrations of Hg^II^ and MeHg from tracers and ambient Hg (Tables S5 and S6); ancillary parameters (Table S7); fraction of Hg occurring as MeHg in the benthic invertebrates (Table S8) (PDF)
Concentrations of Hg^II^ and MeHg from tracers and ambient Hg (Tables S5 and S6) (XLSX)
