# Supplementary material for: Geochemical and Dietary Drivers of Mercury Bioaccumulation in Estuarine Benthic Invertebrates
Source: Environ Sci Technol. 2022 Jun 30;56(14):10141–8. doi: 10.1021/acs.est.2c03265 (PMC9301910; doi:10.1021/acs.est.2c03265)
Supplement: Supplementary file 1 — es2c03265_si_001.pdf [file es2c03265_si_001.pdf]

Supporting Information for

Geochemical and Dietary Drivers of Mercury Bioaccumulation in Estuarine Benthic Invertebrates

Sofi Jonsson, Van Liem-Nguyen, Agneta Andersson, Ulf Skyllberg, Mats B. Nilsson, Erik Lundberg and Erik Björn

Corresponding Author: Sofi Jonsson, sofi.jonsson@aces.su.se

Index

Supplementary methods S1

Figure S1. Map showing sediment sampling location and location for data presented in Table S2 **S2**

Figure S2. MeHg-BSAF and Hg^{II}-BSAF for tracers in M1-NP_{low}, M1-NP_{high}, M1-TM and M2 **S3**

Figure S3. MeHg-BSAF and Hg^{II}-BSAF for ambient Hg in M1-NP_{low}, M1-NP_{high}, M1-TM and M2 **S4**

Figure S4. Percent of carbon and nitrogen content **S4**

Table S1. Pelagic biological productivity parameters **S5**

Table S2. Abundance of benthic invertebrates in 2010 **S5**

Table S3.. Taxonomical information of zoo benthos found close to the sampling site in 2010 **S6**

Table S4. Number of individuals collected and ambient MeHg concentrations **S7**

Table S5 and S6. *MeHg and HgII concentrations in biota and sediment* ***Sep. file***

Table S7. Ancillary parameters from the water column **S8**

Table S8. Fraction of Hg as MeHg (%) in collected invertebrates **S8**

Supplementary methods

Analysis of Hg in biota. Briefly, the isotope selective concentrations of total Hg and MeHg were quantified using isotopically enriched Hg and MeHg as internal standards, (for MeHg) separation of the targeted Hg species using a gas chromatograph (GC) and isotope specific analysis with the help of an Inductively Coupled Plasma Mass Spectrometer (ICPMS). Concentrations of tot-Hg and MeHg for ambient Hg and tracers were then calculated from mass-bias corrected signals using signal deconvolution and Hg^{II} was calculated as the difference between determined MeHg and tot-Hg. The MeHg was extracted from the biota prior to the analysis using 20% (w/w) tetramethylammonium hydroxide in MQ water solution for 2 h in an ultrasonic bath at a temperature of 60 °C[1]. The extracted MeHg was then preconcentrated on a solid absorbent after direct ethylation before the analysis on the GC-ICPMS. Prior to the analysis of total Hg, the biota matrix was digested with ultrapure HNO_3:H_2SO_4 (1:1) at 80 °C over night and then determined using on-line cold vapor generation with $SnCl^2$ reduction connected to an GC-ICPMS.

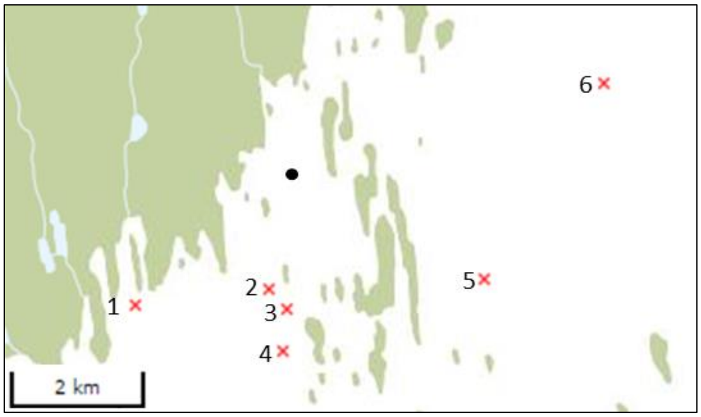

Figure S1. Map showing sediment sampling location for the mesocosm studies (black circle) and locations where the composition of benthic zoobenthos has been characterized (red cross) as shown in Table S2. Map provided by Swedish Meteorological and Hydrological Institute (http://www.smhi.se/klimatdata/oceanografi/havsmiljodata/marina-miljoovervakningsdata).

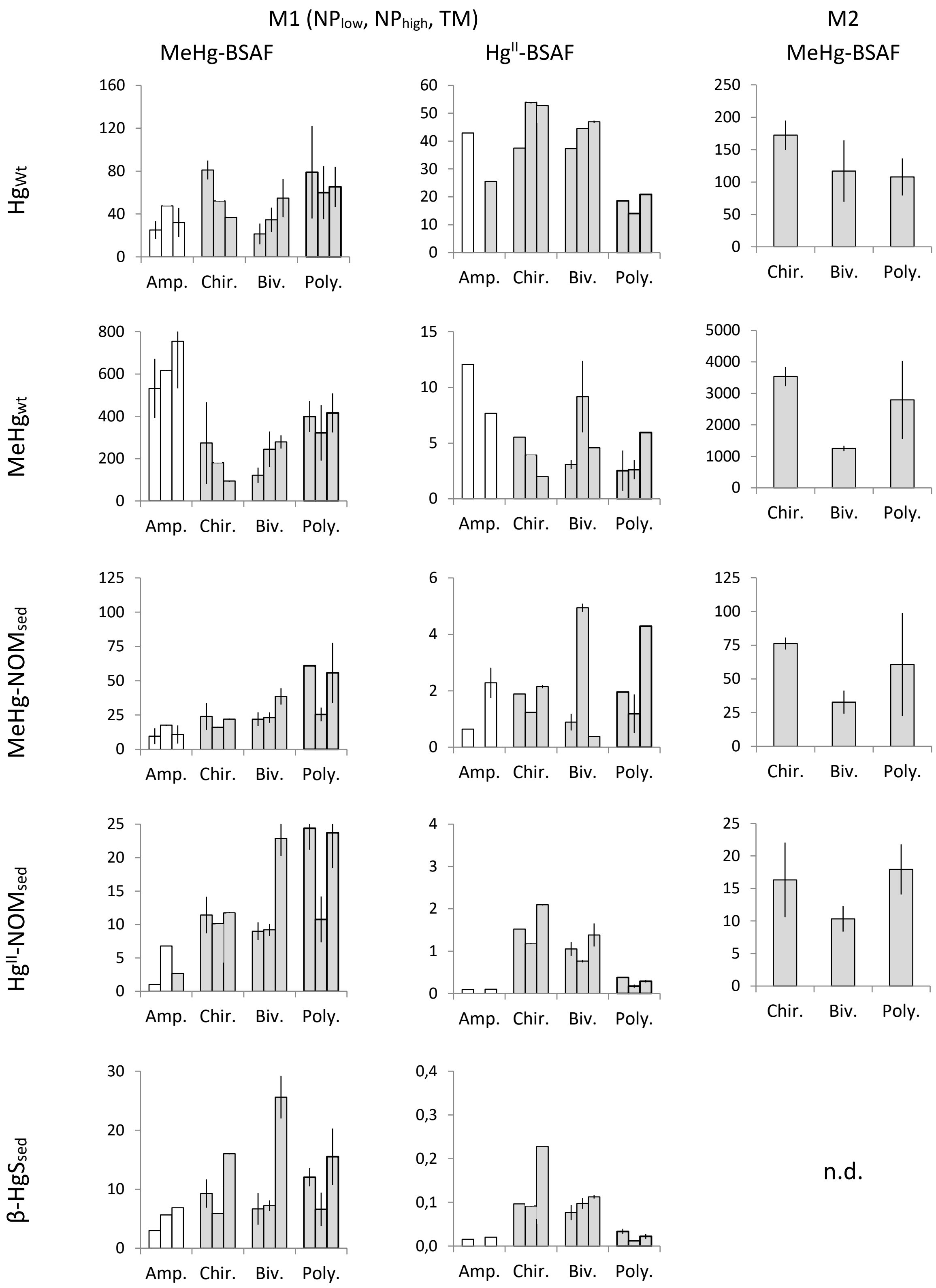

Figure S2. Average MeHg-BSAF and HgII-BSAF (± SE) in ampipods (Amp.), chironomids (Chir.), bivalves (Biv.) and polychates (Poly.) for the five Hg isotope tracers added in mesocosm systems M1 and M2.

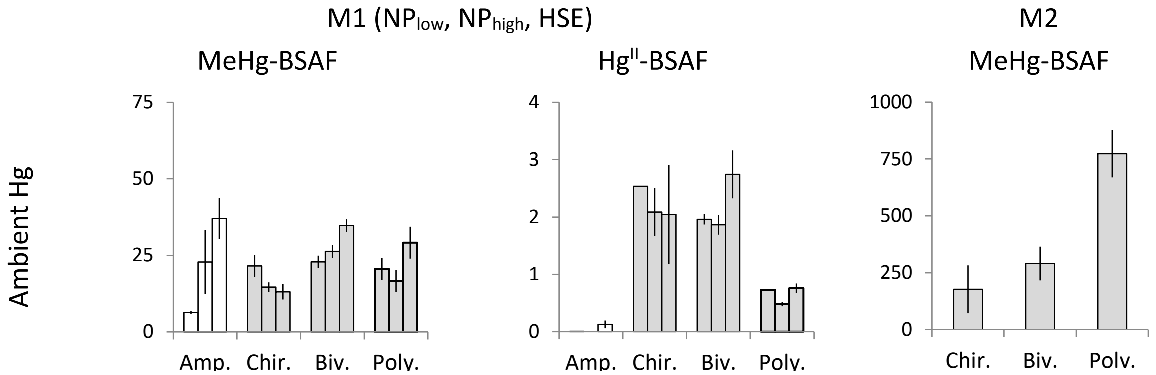

Figure S3. Average MeHg-BSAF and HgII-BSAF (± SE) in ampipods (Amp.), chironomids (Chir.), bivalves (Biv.) and polychates (Poly.) for ambient Hg in mesocosm systems M1 and M2.

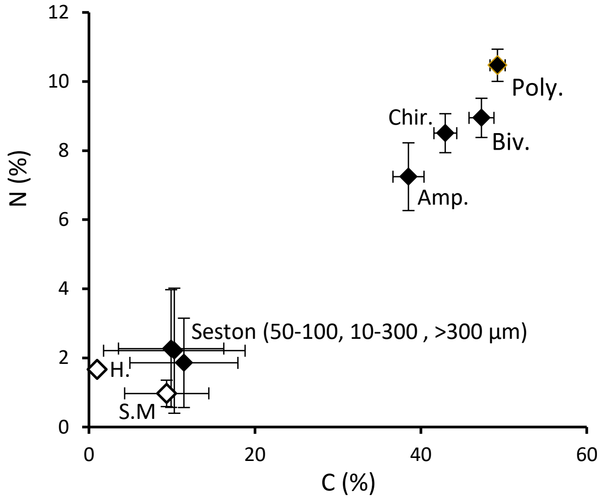

Figure S4. Percent of carbon and nitrogen content in collected seston fractions, benthic invertebrates and sedimenting material (S.M) collected from M1 systems and in terrestrial matter added to M1-TM.

Table S1. Compilation of previously published[2–4] pelagic biological productivity parameters illustrating the key treatment effects (Average (±SE)^n) concentration of Chl a, Primary production and Bacterial Production) in mesocosm experiment M1 and M2 (from day 8) following additions of nutrients or terrestrial humic organic matter.

	Nutrient addition	Chl a mg m^{-3}	Primary production (µmol C dm^{-2} h^{-1})	Bacterial production (µmol C dm^{-2} h^{-1})
M1-NP_{low}[3]	Moderate NP	**5.60** (0.60)	**2.25** (0.25)	**1.94** (0.08)
M1- NP_{high}[2]	High NP	**10.6** (2.2)	**3.21** (0.57)	**2.88** (0.29)
M1-TM[2]	Humic matter	**2.89** (0.20)	**0.97** (0.11)	**2.52** (0.25)
M2[4]	Moderate to high NP	**4.3** (0.51)[27]	**2.9** (0.65)[15]	**4.1** (0.53)[12]

superscript numbers in the table refer to literature references

Table S2. Abundance of benthic invertebrates (individuals m^{-2}) in 2010 at six sites close to the site where the sediment for the M1 and M2 systems were collected (see Figure S1).[1)]

**Station nr.<br>depth (water)**	**1<br>7.9**	**2<br>13.1**	**3<br>8.7**	**4<br>15.3**	**5<br>23**	**6<br>20.4**	**Average (1-6)**
Macoma balthica	-	210	-	10	610	-	**140**
Chironomidae	10	20	-	20	-	-	**8.4**
Monoporeia affinis	20	30	606	20	10	140	**140**
Corophium volutator	-	-	-	20	-	10	**5.1**
Gammarus	20	-	-	-	-	-	**3.4**
Marenzelleria	-	440	-	110	570	810	**320**
Oligochaeta	710	10	40	40	-	-	**130**
Cyanophthalma obscura	-	-	10	-	-	-	**1.7**
Nemata	710	-	-	-	-	-	**120**
Saduria entomon	30	20	10	10	10	10	**15**

1) Data provided by Swedish Meteorological and Hydrological Institute, (http://www.smhi.se/klimatdata/oceanografi/havsmiljodata/marina-miljoovervakningsdata)

Table S3. Taxonomical information of zoo benthos found close to the sampling site (Table S1, Figure S1) in 2010 in the national monitoring program.

Scientific name	Phylum	Class	Order	Family	Genus	Species
**Macoma balthica**	*Mollusca*	*Bivalvia*	*Heterodonta*	*Tellinidae*	*Macoma*	*Macoma balthica*
**Chironomidae**	*Arthro-poda*	*Insecta*	*Diptera*	*Chironomidae*		
**Monoporeia affinis**	*Arthro-poda*	*Malaco-straca*	*Amphipoda*	*Pontopore-iidae*	*Monoporeia*	*Monoporeia affinis*
**Corophium volutator**	*Arthro-poda*	*Malaco-straca*	*Amphipoda*	*Corophidae*	*Corophium*	*Corophium volutator*
**Gammarus**	*Arthro-poda*	*Malaco-straca*	*Amphipoda*	*Gammaridae*	*Gammarus*	
**Marenzelleria**	*Annelida*	*Polychaeta*	*Spionida*	*Spionidae*	*Marenzelleria*	
**Oligochaeta**	*Annelida*	*Clitellata*				
**Cyanophthalma obscura**	*Nemertea*	*Enopla*	*Hoplone-mertea*	*Monostilifera, genera incertae sedis*	*Cyanophthalma*	*Cyanophthalma obscura*
**Nematoda**	*Nematoda*					

[1)] Data provided by Swedish Meteorological and Hydrological Institute, (http://www.smhi.se/klimatdata/oceanografi/havsmiljodata/marina-miljoovervakningsdat)

Table S4. Number of individuals collected (N (ind.)) in the 3 mesocosms and average (SE in italic) ambient MeHg concentrations.

	**M1-NP$_{low}$**			**M1-NP$_{high}$**			**M1-TM**		
	**N (ind.)**	**[MeHg]**		**N (ind.)**	**[MeHg]**		**N (ind.)**	**[MeHg]**	
S. 50-100		**2.2**	*0.75*		**3.9**	*0.51*		**39**	*13*
S.100-300		**5.3**	*3.4*		**4.8**	*1.9*		**54**	*43*
S. >300		**2.4**	*0.89*		**2.9**	*1.1*		**69**	*34*
Amp.	1,2,1	**20**	2.3	1, 2, 0	**60**	*27*	3, 5, 4	**120**	*16*
Chi.	21,1,1	**71**	16	30, 4, 0	**39**	*4.6*	11, 4, 0	**43**	*6.5*
Biv.	44,26,38	**73**	7.1	46, 18, 36	**72**	*6.7*	48, 35, 25	**110**	*9.0*
Poly.	40,19,12	**71**	12	30, 12, 6	**46**	*12*	20, 21, 17	**94**	*15*

	**M2**	
	**[MeHg]**	
S. 50-100	**24**	*2.9*
S.100-200	**29**	*6.0*
S. >200	**56**	*6.0*
Chi.	**190**	*80*
Biv.	**300**	*92*
Poly.	**810**	*38*

Table S7. Average concentrations (CI) throughout the experiment of humic matter (quantified as quinine sulphate units), DOC, average (400, 500, 600 and 700 nm) transmittance (%) and O2 (% saturation) in the water column of M2 at different water depths.

	Water depth (m)	M1-NP_{low}	M1-NP_{high}	M1-TM
Humic Matter (µg L^{-1})	1.5	18 (±0.44)	19 (±0.78)	28 (±0.98)
	4.5	19 (±0.44)	19 (±0.77)	24 (±1.12)
DOC (mg L^{-1})	1.5	5.1 (±0.40)	4.8 (±0.19)	5.7 (±0.42)
	4.5	5.0 (±0.44)	4.7 (±0.40)	5.4 (±0.29)
Transmittance (%)	0.5 or 1.5	98.5 (±0.15)	98.3 (±0.39)	95.6 (±1.47)
	4.5	98.3 (±0.17)	98.5 (±0.25)	97.4 (±0.20)
O2 (% saturation)	1.5	74 (±4.2)	103 (±15)	72 (±6.2)
	4.5	33 (±4.2)	42 (±4.8)	31 (±3.8)

Salinity. pH and DOC is from M1 is available elsewhere (Supplemetnary table S4 in Van et al. 2016)

Table S8. Average (SE in italic) fraction of Hg as MeHg (%) for ambient Hg and added Hg tracers in the benthic invertebrates' amphipods (Amp.), chironomids (Chir.), bivalves (Biv.), and polychaetes (Poly.) in M1 mesocosms (including samples where the concentration of both MeHg or Hg^{II} were >d.l.).

	$Me^{198}Hg$-NOM_{sed}		^{201}Hg-NOM_{sed}		β-$^{200}HgS_{sed}$		$Me^{199}Hg_{wt}$		$^{204}Hg_{Wt}$		Hg_{Amb}	
**Amp.**	**64**	*14.8*	**15**	*1.9*	**13**	*1.6*	**98**	*5.7*	**2.8**	*0.6*	**80**	*8.1*
**Chir.**	**69**	*8.0*	**5.2**	*0.7*	**4.6**	*1.7*	**94**	*24*	**2.2**	*0.2*	**5.1**	*1.3*
**Biv.**	**87**	*5.0*	**8.7**	*1.2*	**5.7**	*1.3*	**79**	*5.1*	**1.7**	*0.3*	**8.6**	*0.5*
**Poly.**	**91**	*4.8*	**35**	*3.0*	**22**	*4.6*	**91**	*3.1*	**7.2**	*0.9*	**20**	*2.2*

References

(1) Qvarnström, J.; Frech, W. Mercury Species Transformations during Sample Pre-Treatment of Biological Tissues Studied by HPLC-ICP-MS. *J. Anal. At. Spectrom.* **2002**, *17* (11), 1486–1491. https://doi.org/10.1039/b205246f.

(2) Jonsson, S.; Andersson, A.; Nilsson, M. B.; Skyllberg, U.; Lundberg, E.; Schaefer, J. K.; Åkerblom, S.; Björn, E. Terrestrial Discharges Mediate Trophic Shifts and Enhance Methylmercury Accumulation in Estuarine Biota. *Sci. Adv.* **2017**, *3* (1). https://doi.org/10.1126/sciadv.1601239.

(3) Jonsson, S.; Skyllberg, U.; Nilsson, M. B.; Lundberg, E.; Andersson, A.; Björn, E. Differentiated Availability of Geochemical Mercury Pools Controls Methylmercury Levels in Estuarine Sediment and Biota. *Nat. Commun.* **2014**, *5*, 4624. https://doi.org/10.1038/ncomms5624.

(4) Nguyen, V. L.; Jonsson, S.; Skyllberg, U.; Nilsson, M. B.; Andersson, A.; Lundberg, E.; Björn, E. Effects of Nutrient Loading and Mercury Chemical Speciation on the Formation and Degradation of Methylmercury in Estuarine Sediment. *Environ. Sci. Technol.* **2016**, *50* (13), 6983–6990. https://doi.org/10.1021/acs.est.6b01567.
